# Design and Production of Geranylated Cyclic Peptides by the RiPP Enzymes SyncM and PirF

**DOI:** 10.1021/acs.biomac.5c00260

**Published:** 2025-04-07

**Authors:** Yanli Xu, Fleur Ruijne, Manel Garcia Diez, Jorrit Jilles Stada, Oscar P. Kuipers

**Affiliations:** Department of Molecular Genetics, Groningen Biomolecular Sciences and Biotechnology Institute, University of Groningen, Groningen 9747 AG, The Netherlands

## Abstract

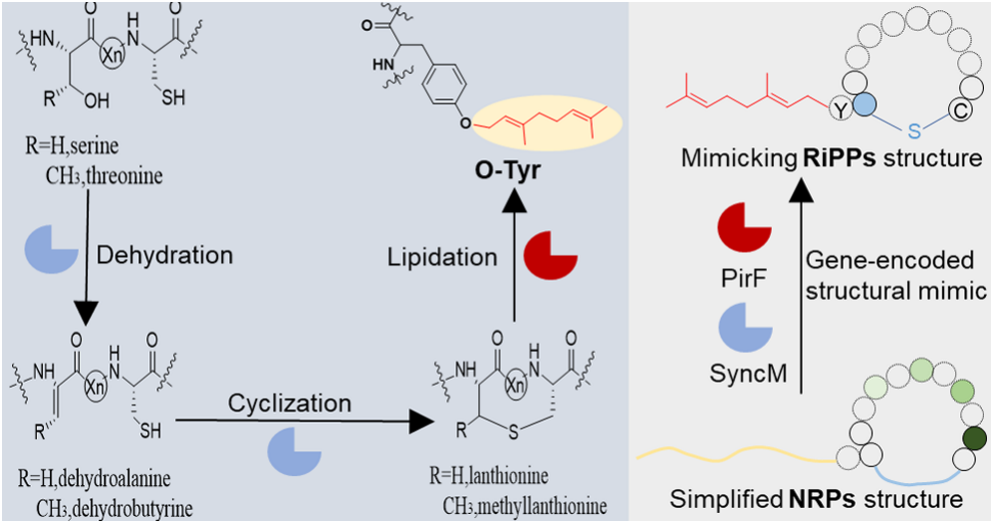

The growing threat of antibiotic resistance highlights the urgent need for new antimicrobial agents. Nonribosomal peptides (NRPs) are potent antibiotics with complex structures, but generating novel NRP analogues is costly and inefficient. An emerging alternative is using ribosomally synthesized and post-translationally modified peptides (RiPPs), which are gene-encoded, allowing for easier mutagenesis and modification. This study aimed to produce peptides with two key structural elements of many NRP antibiotics: a macrocycle and an N-terminal lipid moiety. The RiPP enzymes SyncM and PirF were employed-SyncM introduced lanthionine or methyllanthionine macrocycles, while PirF incorporated isoprenyl chains to emulate the lipid moieties in NRPs. Both enzymes successfully modified the templates, and their combined use generated lipidated macrocyclic peptides, resembling lipopeptide antibiotics. These findings demonstrate the potential of SyncM and PirF as versatile tools for designing novel gene-encoded NRP mimics, enabling high-throughput screening for new bioactive peptides.

## Introduction

The development of novel antimicrobial agents has become increasingly urgent due to the rising threat of antibiotic resistance.^1−5^ Many important antibiotics currently used in the clinic are nonribosomal peptides (NRPs), such as daptomycin and polymyxin B. They are used for the treatment of infections caused by Gram-positive and Gram-negative bacteria, respectively.^6,7^ NRPs form a diverse and structurally complex class of natural products known for their potent and wide-ranging bioactivities, including antibiotic,^6,7^ anticancer,^8,9^ and immunosuppressive effects.^10−12^ NRPs are assembled by large multidomain enzymes called nonribosomal peptide synthetases (NRPSs).^13^ NRPSs operate in a modular fashion, with each module responsible for the incorporation or modification of a specific amino acid into the growing peptide chain.^14−16^ This modularity allows NRPSs to generate peptides with unique structural features, comprising nonproteinogenic amino acids, d-amino acids, lipid tails, and other unusual moieties that contribute to or are essential for their biological activity and stability.^13^ Given the complexity of NRPS-mediated biosynthesis, there is significant interest in understanding and harnessing these pathways to produce novel and improved peptides with tailored biological activities.

Although advances in genetic engineering and synthetic biology have enabled the reprogramming of NRPSs to create NRP variants, current engineering strategies remain limited and technically challenging, highlighting the need for alternative approaches.^17−20^ One such alternative biosynthetic strategy involves leveraging the properties of biosynthetic pathways of ribosomally synthesized and post-translationally modified peptides (RiPPs), a rapidly growing class of natural products that are distinguished by their diverse structures and wide range of biological activities.^21−24^

**Figure 1:**
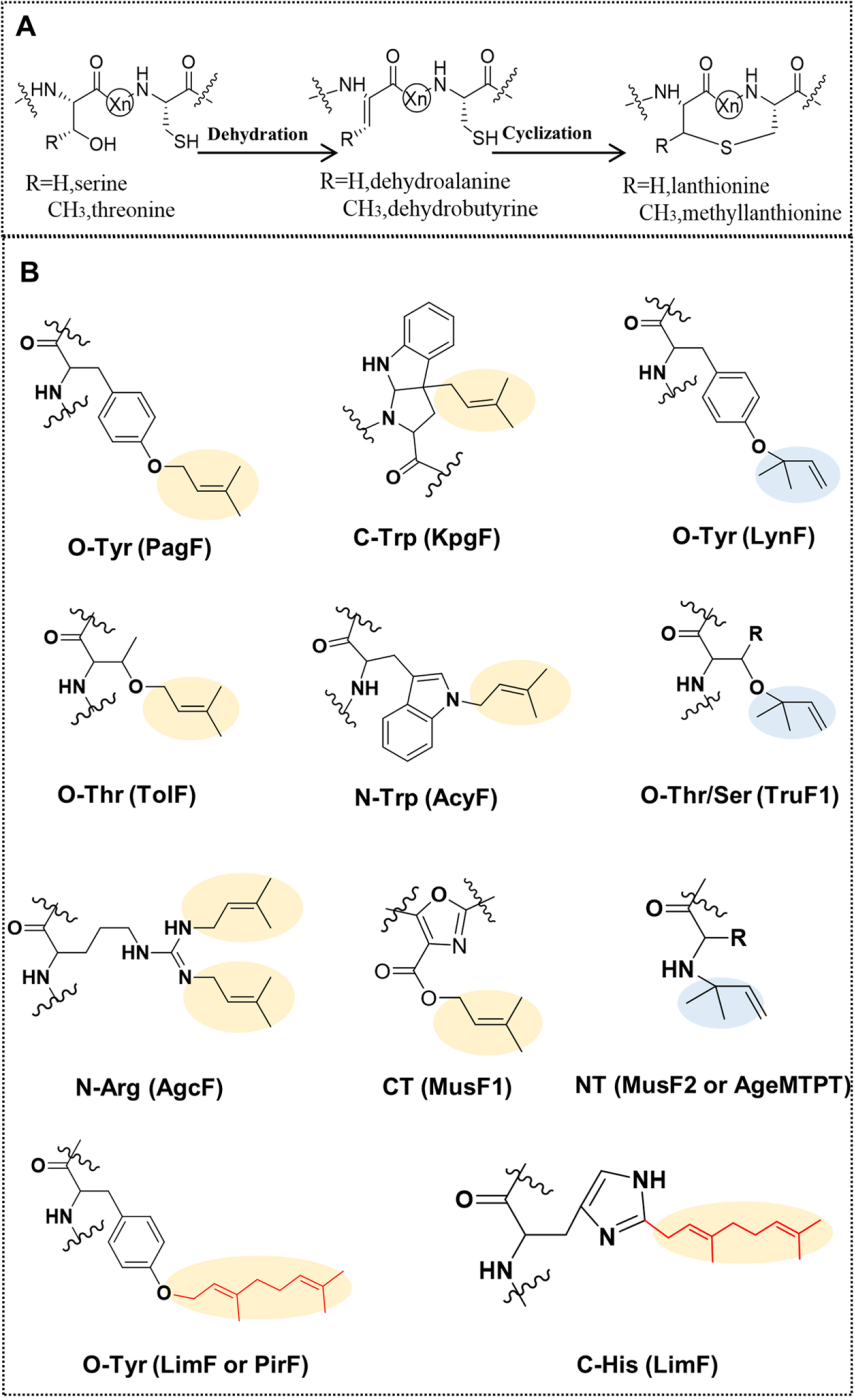
Schematic overview of the general lanthionine biosynthesis pathway and the various chemical groups introduced by cyanobacterial prenyltransferases. (A) The general mechanism of lanthionine biosynthesis, involving the initial dehydration of serine/threonine residues followed by the nucleophilic attack of cysteines on the resulting dehydroamino acids to form (methyl)lanthionine bridges. (B) Types of prenylations catalyzed by cyanobacterial prenyltransferases, with forward prenylation shown in yellow, reverse prenylation in blue, and geranylation marked in red; CT: C terminal; NT: N terminal. PagF,^25^ KpgF,^26^ LynF,^26^ TolF,^27^ AcyF,^28^ TruF1,^29^ AgcF,^30^ MusF1,^27^ LimF,^31^ PirF^32^ are prenyltransferases.

RiPPs are synthesized as precursor peptides, comprising a leader peptide and a core peptide. The leader sequence guides the post-translational modification machinery, ensuring proper processing of the core peptide. The precursor peptides can be extensively modified by a variety of enzymes to yield mature bioactive compounds after leader peptide removal.^33−35^ Among the most studied RiPPs are lanthipeptides, which are characterized by the presence of lanthionine and/or methyllanthionine residues. (Methyl)lanthionines are formed via enzyme-catalyzed dehydration of serines/threonines and subsequent coupling of the formed dehydroamino acids to the thiol group of cysteines^36^ (Figure 1A). These key modifications in lanthipeptide biosynthesis are catalyzed by lanthionine synthetases. In class I enzymes, dehydration and cyclization are performed by separate enzymes, such as NisB and NisC. In contrast, class II synthetases, like the promiscuous bifunctional enzyme SyncM, catalyze both reactions dehydrating serines/threonines and facilitating cyclization. For cyclization of larger rings, NisC has poor catalytic activity^37^ while SyncM has been shown to display a broad substrate specificity, enabling the introduction of macrocycles of various sizes into various peptides *in vivo*.^38,39^

Another ubiquitous modification in RiPPs biosynthesis involves the addition of prenyl groups to the peptide, a process catalyzed by prenyltransferases (PTases) as found in many cyanobacteria.^40,41^ PTases, such as PirF,^32^ LynF^41^ and PagF,^25^ catalyze the reverse or forward addition of prenyl (5-carbon) or geranyl groups (10-carbon) on Ser, Thr, Tyr, His, Arg or Trp residues on cyclic or linear substrates to produce cyanobactins^26,28,30−32,42−45^ (Figure 1B). Although PTases offer great potential for the addition of various hydrophobic moieties onto peptides, their prenylation activity on non-native peptide substrates, especially those with lanthionine structures, remains largely unexplored. Since the selected templates have relatively long fatty acid chains, we chose Geranyl diphosphate (GPP) as the donor to better mimic this structural feature and used the widely adopted PirF for lipid modification.^46^

The enzymatic versatility of RiPP biosynthesis pathways offers a compelling framework for generating NRP-inspired peptides with tailored bioactivities. This study explores a new combinatorial biosynthesis approach leveraging the promiscuous SyncM and PirF to produce cyclic NRP-mimicking peptides that incorporate key structural features of certain nonribosomal lipopeptide antibiotics, such as macrocyclic rings and N-terminal lipid moieties. Besides showing the potential of these enzymes as tools for the modification of a wide variety of peptides, the novel biosynthesis pathway constructed in this study could serve as a platform for the creation and discovery of new bioactive peptides.

## Materials and Methods

### Strains and Materials

All of the oligonucleotide primers in this study were synthesized from Biolegio B.V. (Nijmegen, The Netherlands) and the sequences are listed in Table S2. The plasmid pET15b-*pir*F was offered by Prof. Eric W. Schmidt. The chaperone plasmid pKJE7 was obtained from the chaperone plasmid set, which was purchased from TaKaRa company (https://www.takarabio.com/products/protein-research/expression-vectors-and-systems/protein-folding-kits/chaperone-plasmid-set). The Gibson Assembly Master Mix enzyme was purchased from New England Biolabs. Deoxynucleotides (dNTPs) and Phusion DNA Polymerase were purchased from Thermo Fisher Scientific (Waltham, MA). Amplified DNA was purified using a NucleoSpin Gel and PCR Clean-up kit (Macherey-Nagel). New constructs plasmid DNA were isolated using a NucleoSpin Plasmid EasyPure kit (Macherey-Nagel). All of the plasmid sequences were confirmed by sequencing by Macrogen Europe (Amsterdam, The Netherlands). Chemicals were purchased from Merck unless specified otherwise. Bacto Tryptone, Bacto yeast extract and glycerol were purchased from BOOM B.V. Antibiotics were used at a final concentration of 50 μg/mL for spectinomycin and kanamycin (Merck), and 100 μg/mL for ampicillin (Formedium). IPTG was obtained from ThermoFisher. The *Escherichia coli* Top10 strain was used for all of the cloning work. For expression studies, *E. coli* BL21(DE3) strain was used. Strains were grown in LB broth (Formedium, Norfolk, United Kingdom) at 37 °C, at 220 rpm, or on LB agar (Formedium, Norfolk, United Kingdom), unless specified otherwise.

### Molecular Cloning

For inserting small core peptide sequences into the previously designed pCDFDuet-hybrid leader-containing vector,^47^ primers corresponding to the core peptide sequences that were codon optimized for *E. coli* expression were used as the DNA insert and cloned (Table S1) into the above vector. All plasmid constructs were confirmed by DNA sequencing (Macrogen Europe, Amsterdam, The Netherlands). Primers used in this study are summarized in supplementary Table S2.

### Expression of Precursor Peptides with SyncM

The pRSF-SyncM^47^ plasmid was transformed into competent *E. coli* BL21(DE3) cells and was cotransformed with the constructed pCDFDuet-NRP-mimics plasmids. Transformed cells were plated on LB plates containing the appropriate antibiotics. Single colonies were inoculated in 4 mL LB broth, supplemented with spectinomycin and kanamycin and grown at 37 °C, 220 rpm, overnight. The overnight culture was diluted 1:50 in Terrific Broth (TB: 24 g/L Bacto Yeast extract, 12 g/L Bacto Tryptone, 5 mL/L glycerol, 0.017 M KH_2_PO_4_, 0.072 M K_2_HPO_4_), supplemented with 1:1000 of spectinomycin and kanamycin. Cell cultures were grown at 37 °C, 220 rpm to an OD_600_ of around 1.0. The cultures were cooled on ice, after which protein and peptide expression was induced with 1 mM IPTG (final concentration), and culturing was continued at 18 °C for ∼20 h, 200 rpm.

### Peptide Purification

The cells (from 100 mL culture) were harvested by centrifugation (4 °C, 8500 rpm, 5 min), resuspended in 20 mL lysis buffer (20 mM NaH_2_PO_4_, 300 mM NaCl, 10 mM imidazole, pH 7.4), and lysed by sonication (10 s ON, 10 s OFF, 45–55% amplitude, 10–15 min). The lysate was obtained by centrifugation (4 °C, 10,000 rpm, 30 min) and filtered through 0.45 μm filters. The lysate sample was loaded on an equilibrated Ni-NTA agarose column and mixed well. The resin was washed with 10 CV wash buffer (20 mM NaH_2_PO_4_, 300 mM NaCl, 40 mM imidazole, pH 7.4) and eluted with 5 mL elution buffer (20 mM NaH_2_PO_4_, 300 mM NaCl, 500 mM imidazole, pH 7.4). The sample was desalted through an equilibrated PD-10 desalting column with Sephadex G-25 resin (GE Healthcare) and eluted in 7 mL 50 mM Tris-HCl pH 8.0. Core peptide was released from the His_6_-tagged leader by 1:20 addition of LahT150 protease (containing 1 mM DTT) for 2 h at 37 °C. The LahT150 protease was purified from *E. coli* containing the pETDuet-LahT150 construct according to the protocol described previously by Bobeica and co-workers.^52^ After leader cleavage, core peptide mixtures were centrifuged (4 °C, 10,000*g*, 15 min), filtered through 0.45 μm filters, and further purified with a second equilibrated Ni-NTA agarose column. The sample was loaded, mixed well and the flow-through containing the core peptide was collected directly, followed by addition of 7 mL second His_6_-tag buffer (20 mM Tris, 300 mM NaCl, pH 7.5).

Core peptides were further purified by an open column with C18 resin (Waters), washed with 3 mL 0.1% trifluoroacetic acid (TFA) in acetonitrile (ACN) and equilibrated with 5 mL Milli-Q + 0.1% TFA. After sample loading, the column was washed with 10 mL 15% ACN + 0.1% TFA, and core peptide was eluted with 8 mL 60% ACN + 0.1% TFA and lyophilized. Lyophilized core peptides served as substrates for further lipidation reactions.

### Prenyltransferase PirF Purification

The pKJE7 chaperone plasmid was transformed to competent *E. coli* BL21(DE3) cells first and new competent cells of this strain were made. The plasmid pET15b-*pir*F was transformed into this new competent pKJE7-containing *E. coli* BL21(DE3) strain. One single colony from the cotransformations was inoculated in 4 mL LB broth medium containing 40 μg/mL chloramphenicol and 100 μg/mL ampicillin (Formedium) and grown overnight at 37 °C, 220 rpm. Two mL of this overnight culture was added into 100 mL fresh LB broth medium (1:50), and grown at 37 °C, 220 rpm until the OD_600_ value reached around 0.6. The culture was induced by the addition of 0.25 mM IPTG and 0.2 mM l-arabinose. The culture was grown for 20 h at 18 °C. Next, the induced cells were harvested by centrifugation at 10,000 rpm for 5 min and the supernatant was discarded. The pellets were resuspended in PBS buffer (20 mM, pH = 8.0). The suspended cells were disrupted by sonication for 20 min with an amplitude of 45% (10 s on, and 10 s off). The lysate was obtained by centrifugation at 10,000 g for 15 min. The supernatant was filtered through 0.45 μm filters. The sample was subjected to His-Tag affinity purification using a Ni-NTA agarose column. The effluent was collected and labeled as flow through (sample Ft). After loading the sample, 20 mL of 10 mM imidazole was used for washing, and then different concentrations of imidazole from 20 mM to 150 mM were used to elute to obtain different fractions which were analyzed by SDS–PAGE. Finally, the fractions containing the target protein were further purified and concentrated by a 50 mL Amicon Ultra Centrifugal Filter, (30 kDa MWCO). The control group followed the same protocol as described, except expressing an empty pET15b plasmid instead of pET15b-*pir*F.

### Lipidation Assays *In Vitro*

Concentrated PirF was utilized as a biocatalyst for prenylation assays, with purified NRP mimics serving as substrates. Each enzyme was tested in a 100 μL reaction system, with specific conditions detailed in Table S6. In the control group, the enzyme was replaced with the corresponding boiled PirF at 100 °C for 15 min, while all other conditions remained constant. Reactions were incubated at 37 °C for 2 h, then halted by centrifugation at 12,000*g* for 10 min. The supernatant was transferred to a fresh tube, and 50 μL of 60% acetonitrile was added to resuspend the pellet containing the modified peptide, followed by a second centrifugation. All supernatants were subjected to MALDI-TOF-MS analysis.

### MALDI-TOF Mass Spectrometry

For MALDI-TOF analysis, 1 μL of the sample was applied to the MALDI target plate and allowed to dry. Following this, 1 μL of matrix solution (comprising 5 mg/mL α-cyano-4-hydroxycinnamic acid in 50% acetonitrile with 0.1% trifluoroacetic acid) was layered on top of the dried sample. Matrix-assisted laser desorption/ionization time-of-flight (MALDI-TOF) mass spectrometry was then conducted using a 4800 Plus MALDI TOF/TOF Analyzer (Applied Biosystems) in reflector positive mode.

### Iodoacetamide Assay

To assess the cyclization state of the peptides, free thiols on the digested precursor peptides were reacted with iodoacetamide (IAA). This reaction occurs only if the cysteine-thiol groups are available, indicating that the lanthionine ring has not formed. Prior to the addition of IAA, 10 μL of the desalted precursor peptide fraction (3.5 mL total) post-PD-10 desalting was incubated for 20 h at room temperature with 1 μL of LahT150 protease. Following this incubation, the samples were treated with 2.5 μL of freshly prepared 20 mM TCEP (dissolved in Milli-Q water, resulting in a final concentration of 1 mM TCEP), along with 27.5 μL of Milli-Q water, and allowed to react for 3 h at room temperature. Subsequently, 10 μL of freshly prepared 50 mM IAA (dissolved in 50 mM Tris-HCl, pH 8.0, yielding a final concentration of 10 mM IAA) was added to the reaction mixture, bringing the final volume to 50 μL, which was then incubated in the dark at room temperature for 1 h. Finally, 1 μL of the reaction mixture was applied to a MALDI-TOF target for mass spectrometry analysis. Each IAA addition increases the monoisotopic mass of a peptide by 57.07 Da.

### Antimicrobial Activity Assay by Agar Diffusion

The antimicrobial activity of NRP-mimicking peptides against various bacteria was evaluated on agar plates, including *Bacillus subtilis* 168, *Staphylococcus aureus*, *E. coli* Top10, *Xanthomonas campestris* and *Lactococcus lactis* NZ9000. *L. lactis* was cultured in GM17 medium and the generated peptides were tested for antibacterial activity on GM17 agar plates containing this strain. All other bacterial strains were cultured overnight in LB medium. The antibacterial assays for these strains were conducted on 1.0% Mueller–Hinton Broth (MHB) agar plates. The medium mixture containing the bacterial strain and the agar medium is poured into the plates and the plates were briefly dried using a flame. After that, freeze-dried purified NRP analogues were reconstituted in 100 μL Milli-Q water, of which 20 μL was spotted onto the agar surface, and allowed to dry. The plates were then incubated at 37 °C for approximately 10 h to assess antimicrobial activity.

## Results and Discussion

### Assembling a Novel RiPP Toolbox

**Figure 2:**
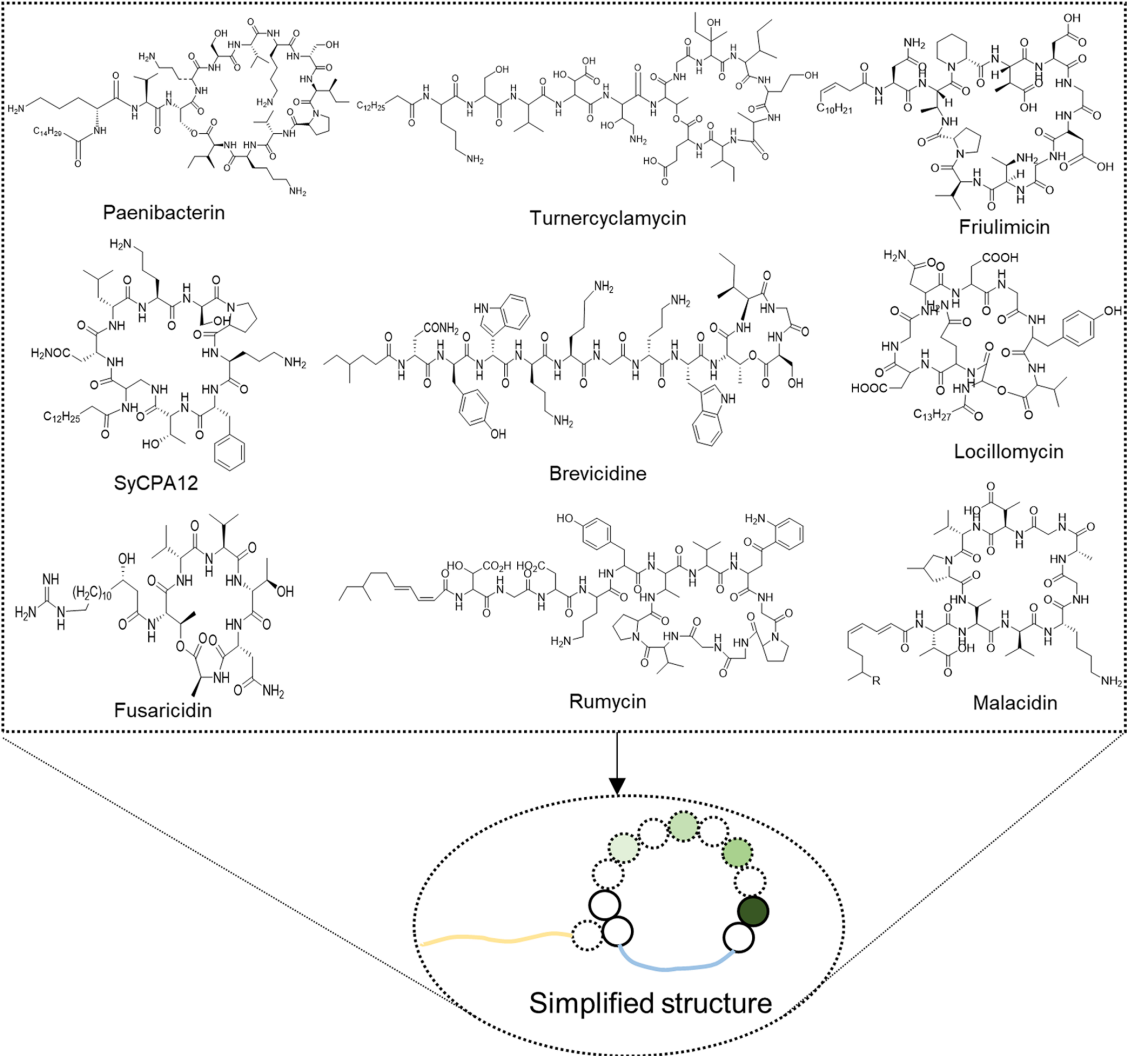
Structures of NRPs selected in this study. The simplified common nonribosomal lipopeptide structure is depicted at the center of the lower ellipse. The small circles represent amino acids; the solid line-circles correspond to the minimum number of ring-forming amino acids of NRP structural mimics generated in this study, while the dotted line circles correspond to possible additional ring-forming amino acids. Nonclassical amino acids, such as ornithine and D-amino acids are depicted in different shades of green. Additional characteristics of these NRPs are shown in Table 1.

In order to establish the successful combinatorial use of RiPP pathways, we first selected a group of cyclic antimicrobial NRP peptide candidates featuring lipid tails of varying lengths and structures and macrocycles of diverse sizes. The chemical structures of these selected structures are depicted in Figure 2 and their key features are summarized in Table 1.

**Table 1:** Summary of Structural Features and Activity of Selected NRPs^a^

nonribosomal peptide	length of lipid tail	macrocycle size (amino acid residues)	other structural features	biological activity
Paenibacterin	C_15_ fatty acyl chain	11	d-amino acids; Ornithines	broad-antimicrobial spectrum
Turnercyclamycin	C_14_ fatty acyl chain	8	d-amino acids; Ornithine	gram-negative pathogens
Friulimicin	C_14_ unsaturated fatty acyl chain	10	diaminobutyric acid (Dab); pipecolinic acid(Pip) and methylaspartic acid (Me-Asp)	gram-positive pathogens
SyCPA12	C_14_ fatty acyl chain	9	d-amino acids; Ornithine	gram-positive bacteria
Brevicidine	C_7_ fatty acyl chain	4	d-amino acids; Ornithines	gram-negative bacteria
Locillomycin	C_13_ fatty acyl chain	9	d-glutamine	antifungal, antibacterial, antivirus
Fusaricidin	15-guanidino-3-hydroxypentadecanoic acid chain	6	d-amino acids	gram-positive bacteria and a wide range of fungi
Rumycin	C_9_ unsaturated fatty acyl chain	9	Kynurenine (Kyn) and hydroxyaspartic acid (hD)	gram-positive pathogens
Malacidin	C_9_ unsaturated fatty acyl chain	9	d-amino acid and five other nonproteinogenic amino acids	gram-positive pathogens

a After selecting these NRP templates to structurally mimic, we assembled the RiPP modification enzymes aimed at introducing macrocycles and lipid moieties.

**Figure 3:**
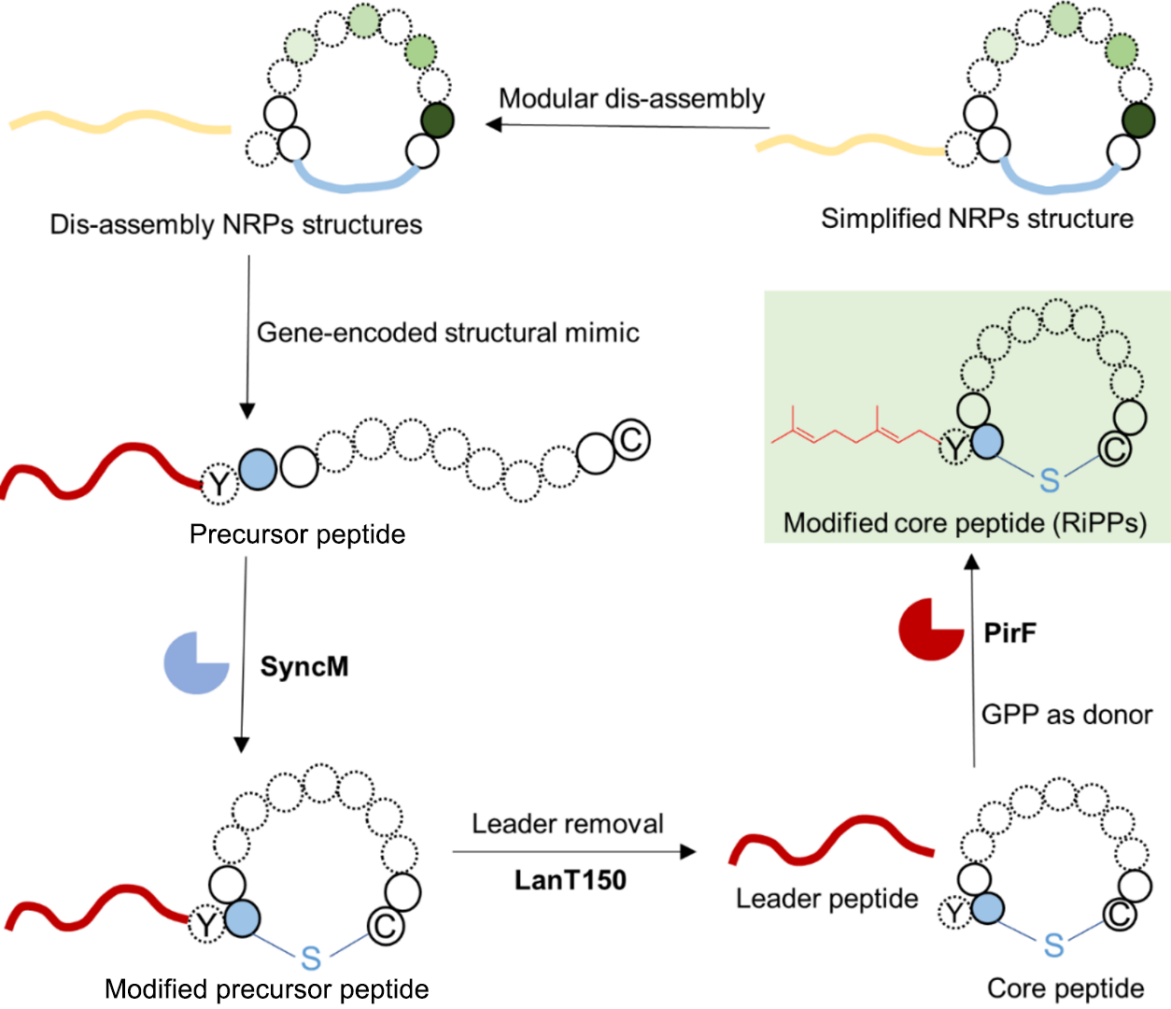
Structural NRP macrocycle and lipid chain mimicking through the combined action of SyncM and PirF. The diagrams illustrate the simplified ring structures and lipid chains that can be introduced in nonribosomal lipopeptide mimicking peptides by the combined action of the RiPP modification enzymes SyncM and PirF. Small circles represent amino acids, with solid lines indicating the amount of amino acid residues present in the smallest macrocycle generated in this study, and dotted lines corresponding to the largest macrocycle. Nonclassical amino acids are shown in varying shades of green, while blue solid circles indicate positions occupied by either threonine (T) or serine (S). The enzyme represented in blue is SyncM, which introduces a (methyl)lanthionine that structurally mimics the macrocycle present in the original nonribosomal lipopeptide. PirF (red enzyme), introduces an isoprenyl chain, mimicking the lipopeptide lipid chain. The background of the final mimicking peptide is depicted in light green. This structural representation facilitates the introduction of targeted mutations and the utilization of enzymes with varying donor lengths for catalysis, enabling the generation of a comprehensive mutant library.

Concerning the introduction of macrocycles, previous work by our group demonstrated that the lanthionine synthetase SyncM can introduce macrocycles of various sizes into several of these selected NRP-inspired peptide templates, given the correct leader peptide sequence was provided.^47^ Lanthionine rings are structurally similar to the macrocycles in the selected NRPs. Furthermore, SyncM has a broad substrate promiscuity and can form diverse macrocycles when a Cys and Ser or Thr are provided in the core peptide sequence.^38^ Therefore, in this study we selected SyncM as the RiPP modification enzyme to introduce macrocyclic structures into our selected nonribosomal lipopeptides (Figure 3).

Next, we selected the RiPP modification enzyme to introduce N-terminal hydrophobic chains into these macrocyclic peptides. While nonribosomal lipopeptides exhibit significant variation in the type and length of the lipid moiety, we hypothesized that the introduction of prenyl and geranyl groups by prenyltransferases could emulate some of the hydrophobic properties conferred by these diverse lipids. Furthermore, in contrast to other known RiPP modification enzymes introducing hydrophobic chains, such as members of the GCN5-related-*N*-acetyltransferase (GNAT) family,^48^ the lipoavitide acyltransferase LpvE,^49^ and the cooperative action of the class III lanthipeptide synthetase MicKC and the cysteine decarboxylase MicD in lipolanthine synthesis,^50^ prenyltransferases appear to be highly promiscuous enzymes, with respect to both peptide substrates and prenyl groups.^27^ In addition, The prenyltransferases from cyanobacteria do not require a leader peptide for core peptide recognition, eliminating the need to design a novel hybrid leader peptide, as is the case for many other hybrid RiPP pathways.^42,51^ To effectively mimic the broad structural diversity of nonribosomal lipopeptides, we therefore selected several prenyltransferases to introduce these hydrophobic chains into the macrocyclic peptides. Among these enzymes, PirF - known for catalyzing the addition of geranyl groups (GPP) and previously demonstrated to facilitate lanthipeptide modification—was selected as the initial test enzyme to explore the potential of prenyltransferases as versatile tools for RiPP lipidation.^43^

### Macrocycle Introduction by SyncM into NRP Mimicking Peptides

Having assembled the RiPP toolkit, we next focused on the design of the peptide substrate. Since many RiPP modification enzymes recognize a leader peptide to install their modifications in the core peptide, we utilized a previously designed short and efficient leader peptide that allows for SyncM recognition and efficient expression in *E. coli*.^47^ Core peptide sequences were designed based on the chosen NRP templates (see Table 1 and Figure 2). To introduce a (methyl)lanthionine ring for mimicking macrocycles in the selected NRP templates, a C-terminal cysteine residue was introduced in the core peptide along with a threonine or serine to allow for lanthionine formation with the desired macrocycle size. D-amino acids were substituted with their corresponding L-forms, and nonstandard amino acids were replaced with structurally similar standard amino acids.^47^ Furthermore, to facilitate N-terminal lipidation, a tyrosine (Y)-based amino terminal N-Tyr-ψ (where ψ is any aliphatic or aromatic residue) recognition motif, derived from PagF -a homologous enzyme to PirF involved in prenylagaramide B biosynthesis^25^- was strategically inserted at the N-terminus of the core peptide to allow for the introduction of a N-terminal hydrophobic chain by various prenyltransferases. The sequences of the resulting NRPs-mimicking core peptides are shown in Table S1.

**Figure 4:**
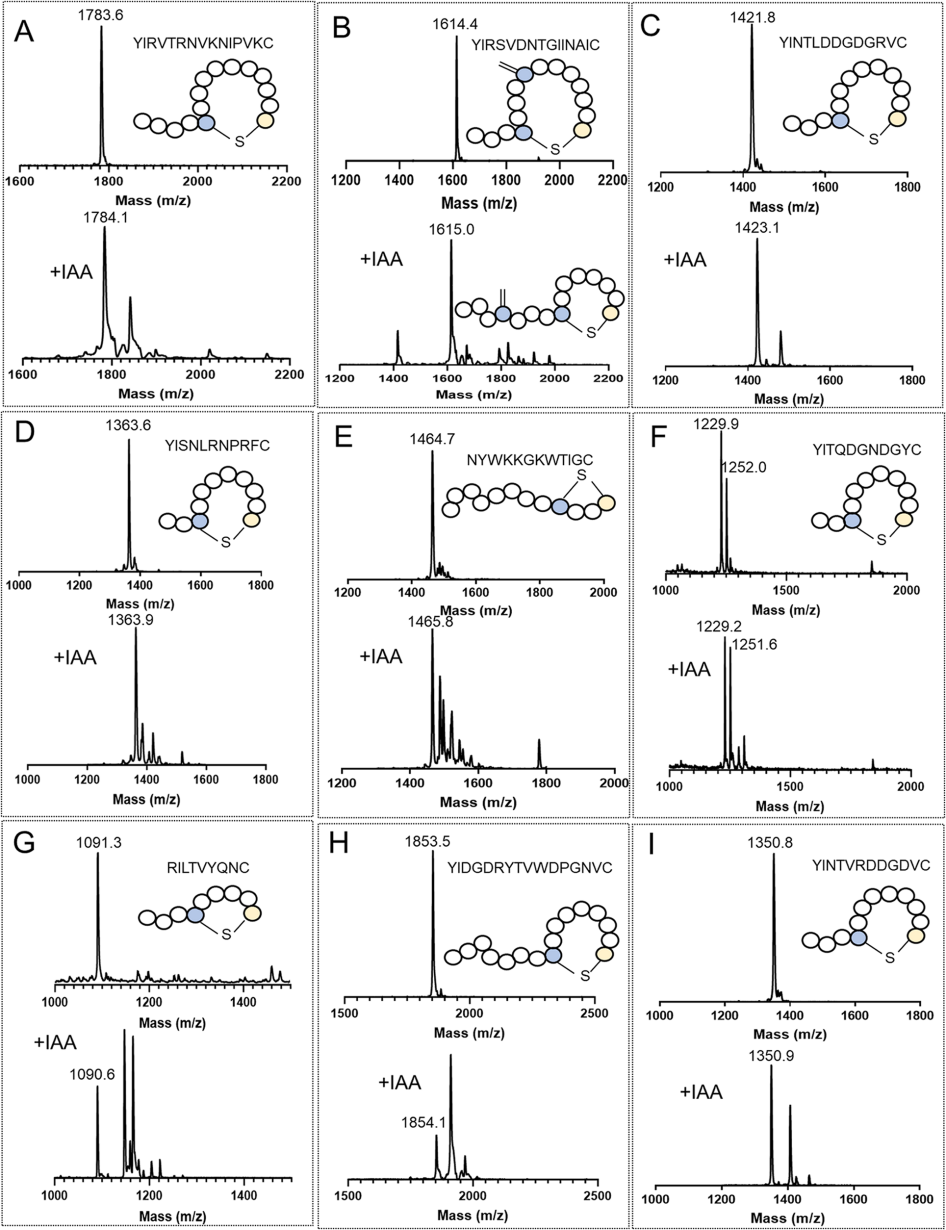
MALDI-TOF MS analysis of dehydration and cyclization catalyzed by SyncM of YI- motif containing peptides. Panels A to I depict the MALDI-TOF mass spectrometry results after dehydration and cyclization by SyncM for various designed NRP mimics. In each box, the upper panel shows the mass spectrometry results of the peptides after SyncM modification, while the lower panel displays the mass spectrometry data obtained after the corresponding iodoacetamide (IAA) treatment (+57 Da), which is used to assess the status of cyclization. (A): Paenibacterin-YI-mimic (YIRVTRNVKNIPVKC); (B): Turnercyclamycin-YI-mimic (YIRSVDNTGIINAIC); (C): Friulimicin-YI-mimic (YINTLDDGDGRVC); (D): SyCPA12-YI-mimic (YISNLRNPRFC); (E): Brevicidine-mimic (NYWKKGKWTIGC); (F): Locillomycin-YI-mimic (YITQDGNDGYC); (G): Fusaricidin-mimic (RILTVYQNC); (H): Rumycin-YI-mimic (YIDGDRYTVWDPGNVC); (I): Malacidin-YI-mimic (YINTVRDDGDVC).

The finalized leader and core peptide constructs were cloned downstream of the IPTG-inducible T7 promoter in the pCDFDuet expression vector, using primers listed in Table S2. Coexpression of these constructs with *syncM* (encoded on a pRSFDuet plasmid) was performed in *E. coli* as described previously.^47^ Post expression, the N-terminally His-tagged peptides were purified and the leader peptide was removed by LahT150,^52^ obtaining the core peptide. Dehydration and lanthionine formation in the core peptide were analyzed by MALDI-TOF mass spectrometry. As shown in Figure 4, all designed peptides were successfully dehydrated. Since lanthionine ring formation after dehydration does not result in a mass difference, iodoacetamide (IAA) assays were subsequently performed to verify the cyclization status (see second panel). Reactivity with IAA indicates the presence of free cysteine residues, indicating dehydration without cyclization.

Gratifyingly, SyncM successfully catalyzed (methyl)lanthionine ring formation across all tested peptides, albeit with varying degrees of efficiency. A summary of masses corresponding to dehydration and cyclization of the core peptides is shown in Table S3.

Of note, the designed Turnercyclamycin-YI-mimic peptide includes the two dehydratable amino acids threonine and serine, along with one cysteine residue for lanthionine formation. As thus only a single lanthionine can be formed, and the IAA cysteine alkylation assay does not provide positional information on the lanthionine ring, two distinct peptide products with differing macrocycle sizes are possible (see Figure 4G, bottom structure displays the desired macrocycle size). Further structural analysis is required to elucidate the specific macrocycle formed. These results confirm SyncM’s previously demonstrated capability to introduce (methyl)lanthionine rings of various sizes, in this study ranging from four to 11 amino acids, into diverse core peptides, regardless of the polarity of the amino acids flanking the serine or threonine residues involved in lanthionine formation.^39^ In summary, SyncM effectively introduced macrocycles in all tested NRP-mimicking peptides. These verified cyclic peptides were used for subsequent lipidation experiments.

### Structural Mimicking of NRP Lipid Tails by PirF

In order to investigate the possibility of using a cyanobacterial prenyltransferase to add a lipid tail to a variety of NRP mimics, we first evaluated the expression of PirF in *E. coli* and *in vitro* activity. The recombinant plasmid pET15b-PirF encoding N-terminally His-tagged PirF was transformed into *E. coli* BL21(DE3) for heterologous expression. Following induction of expression of PirF with isopropyl-β-D-thiogalactoside (IPTG), the majority of the recombinant PirF protein aggregated into inclusion bodies, indicating misfolding of the enzyme. As it is well-established that molecular chaperones can play a crucial role in facilitating proper protein folding and preventing aggregation, to enhance the soluble expression of PirF, coexpression of pET15b-PirF with the chaperone plasmid pKJE7 (from the Takara Chaperone Plasmid Set) was carried out. With the assistance of chaperones, PirF (35 kDa) was successfully expressed in a soluble form in *E. coli*, as can be observed from SDS-PAGE analysis (Figures S1A and S2). Fractions containing the PirF protein were subsequently pooled and concentrated, and the activity of the enzyme was tested using the tripeptide YYY as a substrate and geranyl pyrophosphate (GPP) as the prenyl donor (Sc lane Figure S1A). MALDI-TOF mass spectrometry analysis of the reaction revealed a new peak at 644.04 Da in the reaction group compared to the control (507.50 Da), with the mass difference corresponding to the addition of a single geranyl pyrophosphate donor (approximately 137 Da), thereby confirming active soluble PirF was obtained.

After obtaining active PirF, the previously purified cyclic peptides (Table S1) were incubated with PirF and GPP as the prenyl donor to investigate the activity of PirF on these non-native substrates. Following the *in vitro* reactions, the resulting products were purified using ZipTip and subsequently analyzed by MALDI-TOF-MS. The results are presented in Figure 6.

**Figure 5:**
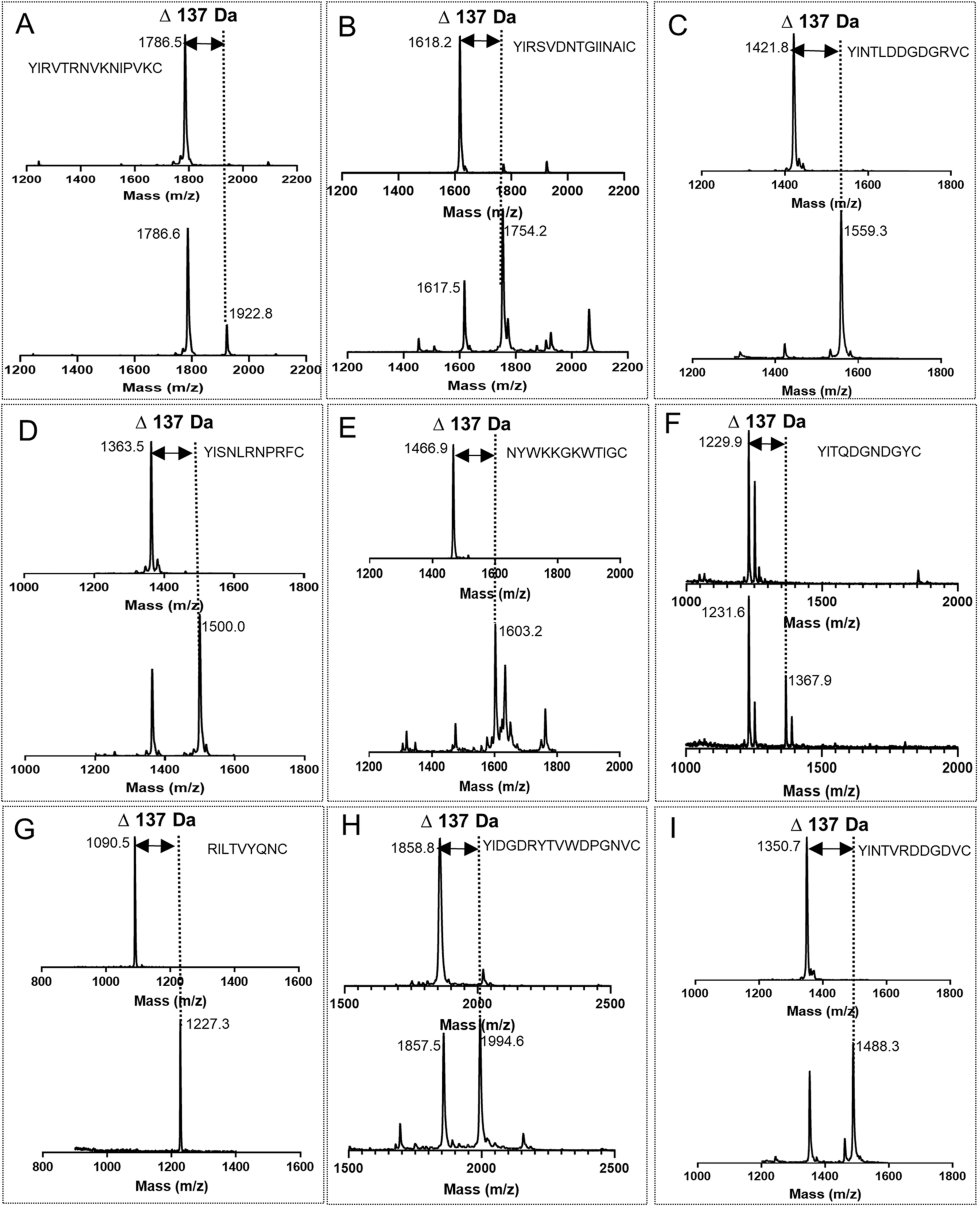
MALDI-TOF MS analysis of the lipidation on the designed Y-containing cyclic peptides by PirF. Panels A to I depict the MALDI-TOF mass spectrometry analysis of lipidation by PirF for various designed Y-containing cyclic NRP mimics. In each box, the upper mass spectrum corresponds to the control group for the lipidation reaction, while the lower spectrum represents the lipidation reaction group. Key mass peaks are highlighted in the Figure. (A): Paenibacterin-YI-mimic (YIRVTRNVKNIPVKC); (B): Turnercyclamycin-YI-mimic (YIRSVDNTGIINAIC); (C): Friulimicin-mimic (YINTLDDGDGRVC); (D): SyCPA12-YI-mimic (YISNLRNPRFC); (E): Brevicidine-mimic (NYWKKGKWTIGC); (F): Locillomycin-YI-mimic (YITQDGNDGYC); (G): Fusaricidin-mimic (RILTVYQNC); (H): Rumycin-YI-mimic (YIDGDRYTVWDPGNVC); (I): Malacidin-YI-mimic (YINTVRDDGDVC).

PirF successfully introduced a geranyl group into various designed peptides containing tyrosine (Y) residues. Although the catalytic efficiency varied, the target molecular weights were clearly detectable in the reaction group compared to the control group (Figure 5). A summary of the relevant mass spectrometry results is provided in Table S5. PirF was able to geranylate Tyr residues at diverse positions within the cyclic peptides, including Tyr residues located at the N-terminus of the designed peptides, as well as Tyr residues located within the linear peptide chain of the brevicidine-mimic, and Tyr residues embedded within the MeLan ring of the Fusaricidin C-mimic (Figure 5, panel G), demonstrating its broad substrate specificity. In the Rumycin-YI-mimic sequences (Figure 5, panel H), a tyrosine residue is present at the N-terminus as well as in the linear region adjacent to the methyllanthionine ring. Conversely, in the Locillomycin-YI-mimic sequences (Figure 5, panel F), the tyrosine residue is not only adjacent to the methyllanthionine ring but also situated within the ring. Although MALDI-TOF mass spectrometry confirms the addition of a single GPP to these substrates, further LC-MS/MS analysis is needed to determine the specific Tyr residue modified, to thereby explore the catalytic preference of PirF. In addition, for the lipidation we employed substrates verified to exist as cyclic peptides, as confirmed by IAA assays. While the IAA analysis demonstrated that most peptides in the samples were cyclic, a small fraction of linear peptides was also detected (Figure 4). The proportion of cyclic versus linear forms varied across samples, implying that lipidation could potentially occur on both forms. Due to the poor water solubility of lipidated peptides, the applicability of IAA assays postlipidation is limited. Nonetheless, based on the proportion of cyclic peptides in the substrate, the hydrophobic nature of the lipidated cyclic peptides, and the enzymatic properties of PirF, we infer that lipidation occurs on both cyclic and linear peptides, though their product ratios differ. Further investigation is required to determine which form predominates for specific peptides. In summary, PirF is capable of introducing lipidation not only at the N-terminal tyrosine, but also along the N-terminal linear sequence. Notably, PirF also facilitates lipidation within a MeLan ring. This versatility highlights PirF as a valuable tool for peptide lipidation.

### Exploration of the Promiscuity of SyncM and PirF

To further validate the promiscuity of SyncM and PirF and explore the potential of combining them to establish a platform for the production of lipidated cyclic peptide products, we designed the following experiments. First, to investigate lanthionine ring formation in a novel context, we modified the design strategy by shifting the macrocycle from the C-terminus to the N-terminus in a peptide based on the NRP brevicidine, thus generating a reversed brevicidine-mimic, named it “brevicidine-mimic mutant” (Table S1). This arrangement did not only remove the negatively charged carboxylate from the brevicidine macrocycle, but also allowed us to explore PirF geranylation on Tyr residues in closer proximity to the C-terminus. Second, to further explore the promiscuity of PirF, we reduced the default YI-motif that was previously introduced in the NRP mimics and recognized by PirF, to a single Tyr residue in the NRP mimics SyCPA12 and Malacidin, resulting in SyCPA12-Y-mimic mutant and Malacidin-Y-mimic mutant (Table S1). This adjustment brings the hydrophobic chain introduced on the Tyr residue by PirF into closer proximity to the macrocycle, thereby more accurately mimicking the structural arrangement observed in their respective nonribosomal peptides.

**Figure 6:**
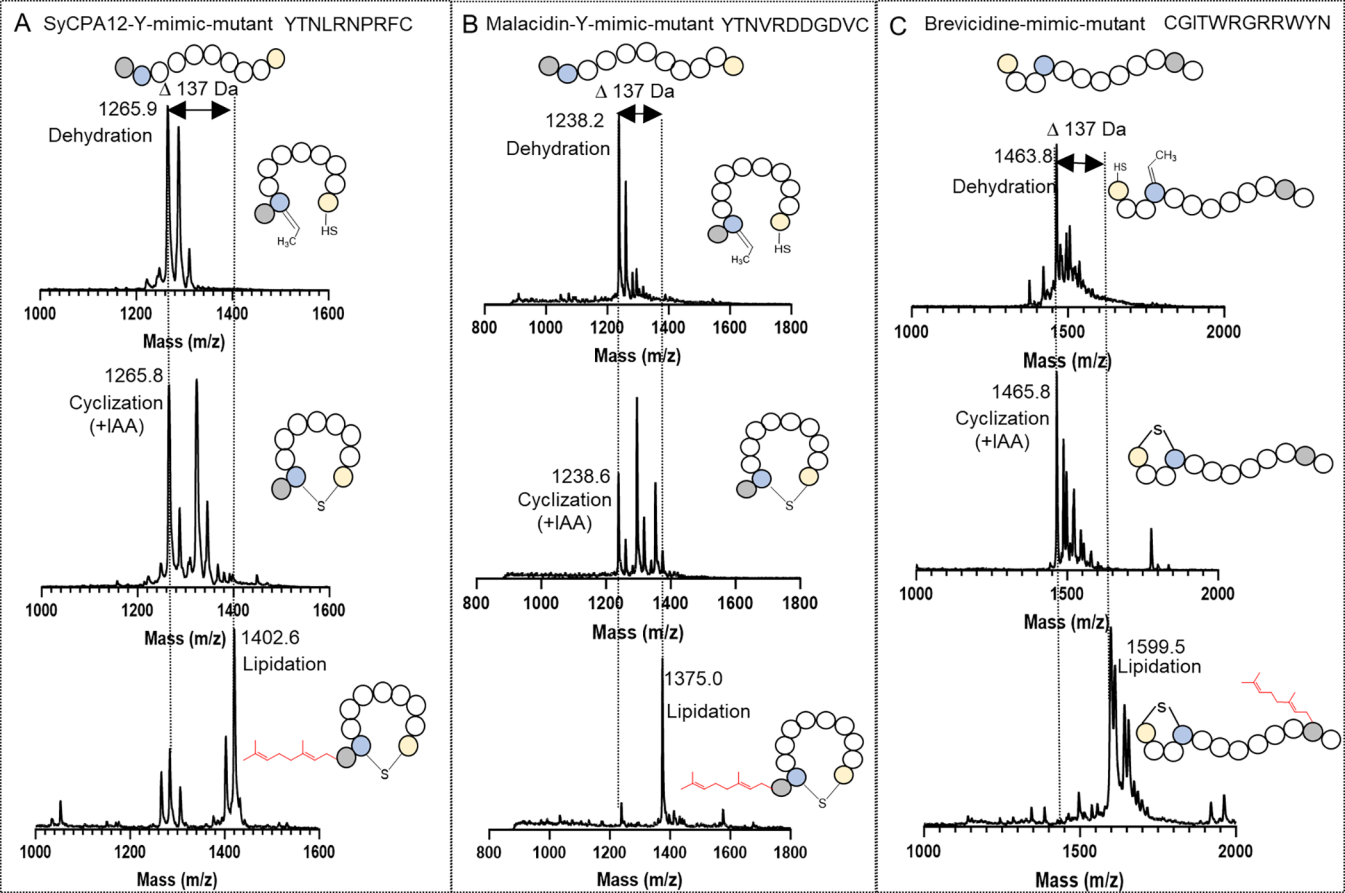
MALDI-TOF-MS analysis of combinations of MeLan formation and lipidation on different mutants catalyzed by SyncM and PirF. Panels A, B, and C depict the MALDI-TOF mass spectrometry analysis of cyclization and lipidation modifications for various mutants. Each box presents the mass spectrometry results in sequence from top to bottom: the dehydration and cyclization products catalyzed by the SyncM enzyme, followed by the lipidation reaction products, the cyclization products were evaluated by IAA reaction. The corresponding simplified structural diagrams and key mass values are included in the Figure. (A): MALDI-TOF-MS analysis of MeLan ring formation and lipidation for SyCPA12-Y-mimic mutant (YTNLRNPRFC) by SyncM and PirF. Top: Dehydration (1265.9). Middle: Cyclization (1265.8). Bottom: Lipidation (1402.6). (B): MALDI-TOF-MS analysis of MeLan ring formation and lipidation for Malacidin-Y-mimic mutant (YTNVRDDGDVC) by SyncM and PirF. Top: Dehydration (1238.2). Middle: Cyclization (1238.6). Bottom: Lipidation (1375.0). (C): MALDI-TOF-MS analysis of MeLan ring formation and lipidation for Brevicidine-mimic mutant (CGITWKGKKWYN) by SyncM and PirF. Top: Dehydration (1463.8). Middle: Cyclization (1465.8). Bottom: Lipidation (1599.5). Blue solid spheres denote threonine residues, gold spheres represent cysteine residues, gray spheres indicate tyrosine residues, and the lipid donor GPP (geranyl group) is depicted in red.

After coexpression of the newly designed peptides with SyncM, MALDI-TOF mass spectrometry analysis revealed that cyclization can successfully occur even when the lanthionine ring is positioned at the N-terminus of the peptide, which is in agreement with previous studies^47^ (Figure 6C). Moreover, the presence of a single tyrosine residue adjacent to the dehydrobutyrine did not impede cyclization (Figure 6A,B). Furthermore, PirF geranylated all novel NRP mimics, demonstrating its ability to modify Tyr residues that are directly adjacent to a lanthionine macrocycle. Furthermore, PirF also modified the C-terminal Tyr residue in the reverse brevicidine mimic, indicating that Tyr residues do not have to be positioned in the N-terminal region for modification by PirF (Table S5). The presence of enzymes like GCN5-related *N*-acetyltransferases,^48^ capable of lipidation *in vivo*, and the broad substrate tolerance of SyncM, provide a foundation for exploring various modification sequences to achieve both cyclization and lipidation *in vivo*. As these are key modifications in therapeutic peptide development, our study integrates peptide backbone synthesis with RiPPs-based enzymatic modifications. This approach offers a promising strategy for the biological production of therapeutic peptides and expands the toolkit for peptide engineering. In conclusion, we show the creation of diverse hybrid RiPP peptides containing (methyl)lanthionines and a geranyl group, by combining the action of SyncM and PirF enzymes that are from different RiPP families. These results lay a solid foundation for the development of additional combinatorial toolboxes for peptide engineering. Furthermore, the generation of nonribosomal cyclic lipopeptide structural mimics opens new avenues for the development of next-generation therapeutics.

### Antimicrobial Activity Assay by Agar Diffusion

In order to investigate whether the generated nonribosomal lipopeptide structural mimics displayed antimicrobial activity, we performed antimicrobial activity agar diffusion assays. We tested the antimicrobial activity of the NRP-mimics against a panel of bacteria, including *B. subtilis* 168, *S. aureus*, *E. coli* Top10, *X. campestris* and *L. lactis* NZ9000. From the generated NRP-mimics in this study, only the SyCPA12 and reverse brevicidine mimics displayed antimicrobial activity (Table S6). We therefore proceeded to test the antimicrobial activity of SyCPA12 and brevicidine mimics in different modification states. Initially, the antimicrobial properties of the NRP mimics containing only a macrocycle (and not a geranyl group) were tested, finding that the brevicidine-mimic mutant and SyCPA12-Y-mimic mutant exhibited antibacterial activity specifically against the Gram-positive bacterium *B. subtilis* 168 (Figure S3A). In contrast, these analogues did not demonstrate any antimicrobial activity against the other bacterial strains tested. In this antibacterial assay, the samples purified are from 200 mL of culture medium for each. Considering when coexpressing SyncM, both linear and cyclic peptides were present in the samples, making the antibacterial results qualitative rather than quantitative due to the incomplete and potentially reversible nature of SyncM modification. To accurately quantify peptide activity, HPLC purification was performed, as shown in the Figures S4 and S5. The HPLC results indicate that brevicidine-mimic mutant eluted at 53.7% ACN, with a low proportion of solely dehydrated (but not cyclized) species (labeled No. 6) while most of the sample consisted of cyclized peptides (No. 7). In contrast, the SyCPA12-Y-mimic mutant sample contained a higher proportion of dehydrated but noncyclized peptides (peak at 1265.9), although the cyclized form remained the dominant product (peak at 1265.8). This difference may be influenced by ring size, as SyCPA12-Y-mimic mutant has a significantly larger ring compared to brevicidine-mimic mutant. Purified products from the same area of HPLC peaks were further tested for antibacterial activity, and the results are shown in Figure S3B. The inhibition zone diameters were measured and summarized in Table 2. The data indicate that both linear and cyclic forms of brevicidine-mimic mutant and SyCPA12-Y-mimic mutant exhibit antibacterial activity. However, the linear form of brevicidine-mimic mutant is more active than its cyclic counterpart, whereas the macrocyclic form of SyCPA12-Y-mimic mutant shows greater activity. This suggests that (Me)Lan ring modification does not necessarily enhance antibacterial activity but rather depends on the specific peptide structure. Given that brevicidine-mimic exhibited activity against the Gram-negative bacterium *X. campestris* when modified by NisBC from class I, but not when processed by SyncM, this suggests structural differences in the core peptide resulting from modifications by these two enzymes.^37^ These structural variations may be attributed to differences in the leader sequence governing peptide folding, the catalytic properties of the modifying enzyme, or the combined influence of both factors.

**Table 2:** Results of Quantitative Test of Antimicrobial Activity

peptides	diameter of inhibition zone (mm)
Brevicidine-mimic mutant (linear)	4
Brevicidine-mimic mutant (cyclic)	8
SyCPA12-Y-mimic mutant-linear (linear)	11
SyCPA12-Y-mimic mutant-linear (cyclic)	6

In the context of the effect of the addition of a geranyl group to the cyclic peptides, lipidation led to a significant decrease in the compound’s solubility, which limited the activity assays to the more soluble lipidated cyclic brevicidine-mimic mutant. Therefore, the antibacterial activity of the lipidated sample was preliminarily assessed using the reaction mixture. As illustrated in Figure S3C, the peptide treated with active PirF demonstrated enhanced antibacterial activity compared to the control, indicating that geranylation might contribute to the improved antimicrobial efficacy of the Brevicidine-mimic mutant. This finding aligns with previous research on similar brevicidine analogues, suggesting lipidation, and in this case more specifically geranylation, enhances antimicrobial activity.^37,53−55^ The absence of activity in the control group could be attributed to the low concentration of the peptide, indirectly suggesting that the overall antibacterial efficacy remains suboptimal and may require further optimization through additional synthetic strategies. In summary, although the observed activity for this lipidated NRP mimic is modest, the increased activity of the brevicidine-mimic mutant calls for further investigation.

For the cyclization of the characteristic thioether cross-links, we used the highly versatile enzyme SyncM in this project. Its broad substrate scope is supported by its diverse natural substrates as well as its ability to catalyze non-natural substrates, as demonstrated in our experiment. However, despite its flexibility, SyncM still has limitations—for instance, it cannot cyclize the highly polar Murepavadin sequence. This highlights the need to further investigate its catalytic mechanism and substrate specificity for the rational design of peptides in the future. Currently, studies on the evolution of SyncM are limited, but extensive research has been conducted on its homologous enzymes, such as ProcM,^56,57^ LctM^58^ and others.^59−61^ These studies have explored their catalytic cyclization domains and substrate preferences, providing valuable insights that could guide the directed evolution of SyncM. Additionally, given that RiPPs undergo multiple cyclization processes, it would be valuable to explore other cyclases in the RiPPs family to better mimic the cyclic structure of the template peptide—particularly those involved in disulfide bond-based cyclization.

For peptide lipidation, we selected PirF, an enzyme that modifies tyrosine with a 10-carbon geranyl moiety. Studies have shown that a single amino acid change can enable PirF and PagF to selectively switch between DMAPP and GPP as donor substrates.^43^ Currently, prenyltransferases from cyanobacteria can modify not only serine, threonine, tyrosine, and tryptophan but also arginine and histidine.^26,30−32,43,62,63^ Cyanobactin prenyltransferases (PTases) belong to the ABBA-fold crystal structure and commonly adopt a α/β barrel fold.^27^ In the biosynthetic pathways of natural products involving these enzymes, lipidation typically occurs as the final step after the leader peptide is cleaved.^42^ This might suggest a broad substrate scope for this enzyme family. However, this also means that when modifying non-natural substrates, *in vitro* catalytic modification is required. Unlike in bacterial systems, where the donor molecule is naturally available, the reaction system must be supplemented with the donor part, increasing experimental costs. Additionally, the donor specificity of these enzymes is limited to DMAPP and GPP, preventing recognition of other chain lengths such as C4 or C8. This limitation makes it difficult to precisely mimic fatty acid chain lengths. A potential solution to these challenges lies in a class of enzymes from the GCN5-related N-acetyltransferase (GNAT) superfamily. Maturases in this group can selectively utilize C10, C12, or C16 fatty acyl groups to modify (hydroxy)ornithine or lysine side chains *in vivo*.^48^ The potential applications of these enzymes in mimicking nonribosomal peptides (NRPs) warrant further investigation.

## Conclusions

In conclusion, the ability of SyncM to successfully form rings of varying sizes, coupled with the effective geranylation by PirF of tyrosine residues located either in linear peptides at the N-terminus, or within methyllanthionine rings, highlights the broad substrate specificity of both enzymes, and their potential as versatile tools in combinatorial RiPP modification toolboxes. Moreover, the successful combined action of SyncM and PirF, enzymes from distinct RiPP families, to generate macrocyclic lipidated peptides, demonstrates the feasibility of creating structural nonribosomal lipopeptide analogues through a hybrid RiPP modification platform. In particular, the broad substrate specificity of PirF as a RiPP lipidation tool sets it apart from the various other RiPP lipidation enzymes discovered until now, since only a tyrosine residue is required in the core peptide sequence to ensure lipidation. Although the lipid donor for PirF is limited to geranyl pyrophosphate, PirF is an interesting tool for the addition of large hydrophobic moieties to peptides, to modulate their hydrophobicity and associated pharmacokinetics. This platform thus offers a valuable approach for generating libraries of structurally diverse peptide analogues, providing a platform for facile activity screening and the discovery and development of novel bioactive peptides.

## Supporting Information Available

Overview of the designed core peptide sequences; overview of primers used; overview of mass summary of modifications; reaction system used for different prenyltransferases in this study; antimicrobial activity assay; HPLC spectra for purification; nucleotide and corresponding amino acid sequences of *pir*F (PDF)
