# Supplementary material for: Design and Production of Geranylated Cyclic Peptides by the RiPP Enzymes SyncM and PirF
Source: Biomacromolecules. 2025 Apr 7;26(5):3186–99. doi: 10.1021/acs.biomac.5c00260 (PMC12076493; doi:10.1021/acs.biomac.5c00260)
Supplement: Supplementary file 1 — bm5c00260_si_001.pdf [file bm5c00260_si_001.pdf]

Supporting Information

Design and Generation of geranylated cyclic NRP-mimics peptides by the RiPP enzymes SyncM and PirF

Yanli Xu, Fleur Ruijne, Manel Garcia Diez, Jorrit Jilles Stada, Oscar P. Kuipers*

Department of Molecular Genetics, Groningen Biomolecular Sciences and Biotechnology Institute, University of Groningen, Groningen, 9747 AG, The Netherlands.

*Correspondence: o.p.kuipers@rug.nl (Oscar P. Kuipers)

Table S1 Summary of designed core peptide sequences of different NRPs-mimics generated in this study. All core peptide sequences were placed behind the hybrid leader designed previously in our group[43].

Name of original NRPs	Designed core sequence	The designated name in this study
Paenibacterin	YIRVTRNVKNIPVKC	Paenibacterin-YI-mimic
Turnercyclamycin	YIRSVDNTGIINAIC	Turnercyclamycin-YI-mimic
Friulimicin	YINTLDDGDGRVC	Friulimicin-YI-mimic
SyCPA12	YISNLRNPRFC	SyCPA12-YI-mimic
Brevicidine	NYWKKGKWTIGC	Brevicidine-mimic
Locillomycin	YITQDGNDGYC	Locillomycin-YI-mimic
Fusaricidin C	RILTVYQNC	Fusaricidin C-mimic
Rumycin	YIDGDRYTVWDPGNVC	Rumycin-YI-mimic
Malacidin	YTNVRDDGDVC	Malacidin-Y-mimic-mutant
Brevicidine	CGITWRGRRWYN	Brevicidine-mimic-mutant
SyCPA12	YTNLRNPRFC	SyCPA12-Y-mimic-mutant
Malacidin	YTNVRDDGDVC	Malacidin-Y-mimic-mutant

Green indicates the position of Ser, Thr or Cys to form a Lan or MeLan.

Red indicates the tyrosine residue to be lipidated.

Table S2 Primer design of different peptides with a hybrid leader attached.

Primer	Sequences (5' to 3')	Purpose
Y-1-ProcM-Fw	CCTGCGTAACCCGCGTTTCTGCTAAGCTTGCGGCCG CATAATGCTTAAGTCG	SyCPA12-Y-mimic construct
Y-2-ProcM-Fw	GTTCGTGACGACGGTGACGTTTGCTAAGCTTGCGGC CGCATAATGCTTAAGTCG	Malacidin-Y-mimic construct
Y-3-ProcM-Fw	GGACGACGGTGACGGTCGTGTTTGCTAAGCTTGCGG CCGCATAATGCTTAAGTCG	Friulimicin-Y-mimic construct
Y-4-ProcM-Fw	GTAACGTTAAAAACATCCCGGTTAAATGCTAAGCTTG CGGCCGCATAATGCTTAAGTCG	Paenibacterin-Y-mimic construct
Y-5-ProcM-Fw	GACAACACCGGTATCATCAACGCTATCTGCTAAGCTT GCGGCCGCATAATGCTTAAGTCG	Turnercyclamycin-Y-mimic construct
Y-6-ProcM-Fw	TACACCGTTTGGGACCCGGGTAACGTTTGCTAAGCT TGCGGCCGCATAATGCTTAAGTCG	Rumycin-Y-mimic construct
Y-7-ProcM-Fw	AGGACGGTAACGACGGTTACTGCTAAGCTTGCGGCC GCATAATGCTTAAGTCG	Locillomycin-Y-mimic construct
YI-1-ProcM-Rv	CGCGGGTTACGCAGGTTAGAGATGTAGCCACCGCTG GCTGCTTCCAGTTC	SyCPA12-Y-mimic construct
YI-2-ProcM-Rv	CACCGTCGTCACGAACGGTGTTGATGTAGCCACCGC TGGCTGCTTCCAGTTC	Malacidin-Y-mimic construct
YI-3-ProcM-Rv	CCGTCACCGTCGTCCAGGGTGTTGATGTAGCCACCG CTGGCTGCTTCCAGTTC	Friulimicin-Y-mimic construct
YI-4-ProcM-Rv	GGGATGTTTTTAACGTTACGGGTAACACGGATGTAG CCACCGCTGGCTGCTTCCAGTTC	Paenibacterin-Y-mimic construct
YI-5-ProcM-Rv	TGATGATACCGGTGTTGTCAACAGAACGGATGTAGC CACCGCTGGCTGCTTCCAGTTC	Turnercyclamycin-Y-mimic construct
YI-6-ProcM-Rv	CCGGGTCCCAAACGGTGTAACGGTCACCGTCGATGT AGCCACCGCTGGCTGCTTCCAGTTC	Rumycin-Y-mimic
YI-7-ProcM-Rv	GTAACCGTCGTTACCGTCCTGGGTGATGTAGCCACC GCTGGCTGCTTCCAGTTC	Locillomycin-Y-mimic construct
Fusaricidin C1-F-ProcM	CTGACCGTTTACCAGAACTGCTAAGCTTGCGGCCGC ATAATGCTTAAGTCG	Fusaricidin C-mimic construct
Fusaricidin C1-R-ProcM	TCTGGTAAACGGTCAGGATACGGCCACCGCTGGCTG CTTCCAGTTCTTC	Fusaricidin C-mimic construct
Bre-procM-Fw	GAAAAAAGGTAAATGGACCATCGGTTGCTAAGCTTG CGGCCGCATAATGCTTAAGTC	Brevicidine-mimic construct
bre- lat-ProcM-Rv	CATTTACCTTTTTTCCAGTAGTTGCCACCGCTGGCTG CTTCCAGTT	Brevicidine-mimic construct
Brevicidine-mimic-mutant-Fw	TGGAAAGGTAAAAAATGGTACAACTAAGCTTGCGGC CGCATAATGCTTAAGTCG	Brevicidine-mimic-mutant construct
Brevicidine-mimic-mutant-Rv	CATTTTTTACCTTTCCAGGTGATACCGCAGCCACCGC TGGCTGCTTCCAGTTCTTC	Brevicidine-mimic-mutant construct
SyCPA12-Y-mimic-mutant-Fw	CTGCGTAACCCGCGTTTCTGCTAAGCTTGCGGCCGC ATAATGCTTAAGTCG	SyCPA12-Y-mimic-mutant construct
SyCPA12-Y-mimic-mutant-Rv	AGAAACGCGGGTTACGCAGGTTGGTGTAGCCACCGC TGGCTGCTTCCAGTTCTTC	SyCPA12-Y-mimic-mutant construct
Malacidin-Y-mimic-mutant-Fw	GTTCGTGACGACGGTGACGTTTGCTAAGCTTGCGGC CGCATAATGCTTAAGTCG	Malacidin-Y-mimic-mutant construct
Malacidin-Y-mimic-mutant-Rv	ACGTCACCGTCGTCACGAACGTTGGTGTAGCCACCG CTGGCTGCTTCCAGTTCTTC	Malacidin-Y-mimic-mutant construct
pRSF _T71_Seq_Fw	TCACCACCCTGAATTGACTC	Sequencing

Table S3 Mass summary of dehydration and cyclization on different core peptides. Mass of cyclization indicated the expected mass of the peptide upon reaction with the cysteine alkylating agent iodoacetamide (IAA), indicating a free Cys residue is present and no cyclization is observed. The column Observed mass indicates the mass observed after reaction with IAA.

Peptide	Core sequence	Mass of core peptide	Mass of Dehydration	Mass of Cyclization	Observed Mass (+IAA)
Paenibacterin-YI-mimic	YIRVTRNVKNIPVKC	1803.20	1785.20	1842.20	1784.17
Turnercyclamycin-YI-mimic	YIRSVDNTGIINAIC	1651.90	1615.90	1672.90	1615.08
Friulimicin-YI-mimic	YINTLDDGDGRVC	1440.55	1422.55	1479.55	1423.14
SyCPA12-YI-mimic	YISNLRNPRFC	1382.60	1364.60	1421.60	1363.94
Brevicidine-mimic	NYWKKGKWTIGC	1483.75	1465.75	1522.75	1465.82
Locillomycin-YI-mimic	YITQDGNDGYC	1248.29	1230.29	1287.29	1229.26
Fusaricidin C-mimic	RILTVYQNC	1109.31	1091.31	1148.31	1090.63
Rumycin-YI-mimic	YIDGDRYTVWDPGNVC	1873.03	1855.03	1912.03	1854.13
Malacidin-YI-mimic	YINTVRDDGDVC	1369.47	1351.47	1408.47	1350.92
SyCPA12-Y-mimic-mutant	YTNLRNPRFC	1283.47	1265.47	1322.47	1265.89
Malacidin-Y-mimic-mutant	YTNVRDDGDVC	1256.31	1238.31	1295.31	1238.65
Brevicidine-mimic-mutant	CGITWRGRRWYN	1483.75	1465.75	1522.75	1465.82

Green indicates the Ser, Thr and Cys residues involved in Lan or MeLan formation.

Table S4 Mass summary on the tripeptide YYY used as an indicator for testing the activity of PirF. Reaction conditions are shown in Table S6.

Enzyme	Peptides	Expected Mass	Observed Mass
PirF	YYY	507.54	507.50
	Modified YYY	644.54	644.04

Table S5 Mass summary of lipidation on different core peptides by PirF.

Peptide	Core sequence	Lipidation control	Expected Mass for lipidation	Observed Mass for lipidation
Paenibacterin-YI-mimic	YIRVTRNVKNIPVKC	1785.20	1922.20	1922.89
Turnercyclamycin-YI-mimic	YIRSVDNTGIINAIC	1615.90	1752.90	1754.27
Friulimicin-YI-mimic	YINTLDDGDGRVC	1422.55	1559.55	1559.35
SyCPA12-YI-mimic	YISNLRNPRFC	1364.60	1501.60	1500.03
Brevicidine-mimic	NYWKKGKWTIGC	1465.75	1602.48	1603.18
Locillomycin-YI-mimic	YITQDGNDGYC	1230.29	1367.29	1367.97
Fusaricidin C-mimic	RILTVYQNC	1091.31	1228.31	1227.33
Rumycin-YI-mimic	YIDGDRYTVWDPGNVC	1855.03	1992.03	1994.61
Malacidin-YI-mimic	YINTVRDDGDVC	1351.47	1488.47	1488.38
SyCPA12-Y-mimic-mutant	YTNLRNPRFC	1265.47	1402.47	1402.64
Malacidin-Y-mimic-mutant	YTNVRDDGDVC	1238.31	1375.31	1375.08
Brevicidine-mimic-mutant	CGITWRGRRWYN	1465.75	1602.75	1599.55

Red amino acid indicates the tyrosine residue to be lipidated.

Table S6 Antibacterial activity screening results of different purified peptides in this study.

Strains / Peptides	*Bacillus subtilis* 168	*Staphylococcus aureus*	*Escherichia coli* Top10	*Xanthomonas campestris*	*Lactococcus lactis* NZ9000
Paenibacterin-YI-mimic	-	-	-	-	-
Turnercyclamycin-YI-mimic	-	-	-	-	-
Friulimicin-YI-mimic	-	-	-	-	-
SyCPA12-YI-mimic	√	-	-	-	-
Brevicidine-mimic	√	-	-	-	-
Locillomycin-YI-mimic	-	-	-	-	-
Fusaricidin C-mimic	-	-	-	-	-
Rumycin-YI-mimic	-	-	-	-	-
Malacidin-YI-mimic	-	-	-	-	-
SyCPA12-Y-mimic-mutant	√	-	-	-	-
Malacidin-Y-mimic-mutant	-	-	-	-	-
Brevicidine-mimic-mutant	√	-	-	-	-

Table S7. Prenylation reaction conditions used for testing the activity of PirF in this study.

Compounds	Volume (μL)
Bis-tris-propane (pH=7.5, 500 mM)	10
$MgCl_2$ (1.0 M)	10
NaCl (1.0 M)	10
GPP (10 mM)	10
YYY (10 mM)	10
Milli-Q	30
PirF enzyme	20

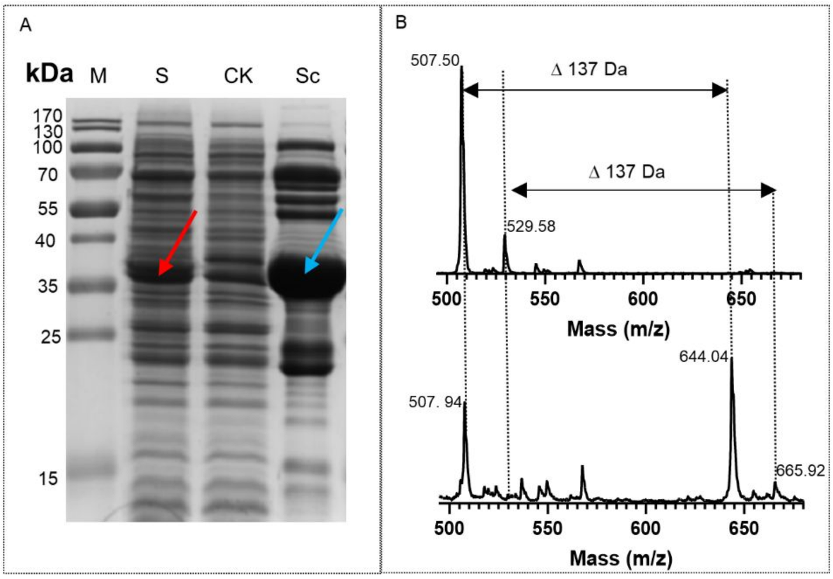

Figure S1. SDS-PAGE and mass spectrometry analysis of heterologous expression and activity of the PirF prenyltransferase.

(A) SDS-PAGE analysis of heterologous expression, purification and concentration of PirF co-expressed with chaperone proteins in *E.coli* BL21(DE3). M: Marker, S: total protein of *E.coli* BL21(DE3) [pET15b-pirF] in the supernatant fraction; CK: total protein of the control strain *E.coli* BL21(DE3) [pET15b] in the supernatant fraction; Sc: concentrated PirF, Red and blue arrows show soluble recombinant PirF is produced (35 kDa). (B) PirF activity test using YYY as the substrate and GPP as the prenyl donor. The upper panel shows the control group with boiled PirF, while the B lower panel shows the test group with the active PirF enzyme. The theoretical mass of the tripeptide YYY is calculated to be 507.54 Da, with a predicted mass of 644.54 Da following geranylation. The experimentally observed values are consistent with these predictions, yielding 507.50 Da for the unmodified tripeptide and 644.04 Da for the Tyr-geranylated product (Table S4).

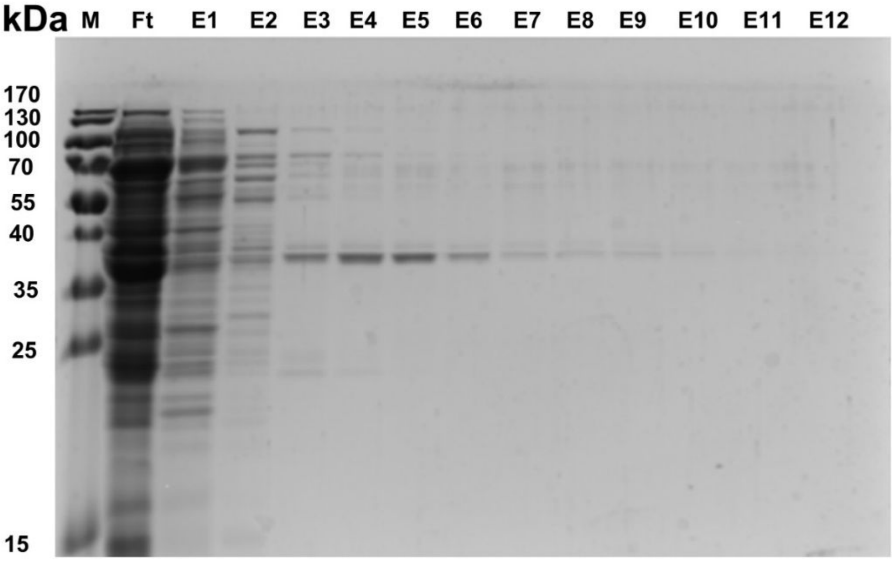

Figure S2. SDS-PAGE analysis of different fractions of PirF after Ni-NTA purification with varying imidazole concentrations. "Ft" represents the flow through after the His$_6$-tag column, while E1 through E12 correspond to different elution buffers containing various imidazole concentrations of 10 mM, 20 mM, 30 mM, 40 mM, 50 mM, 60 mM, 70 mM, 80 mM, 90 mM, 100 mM, 120 mM, and 150 mM, respectively.

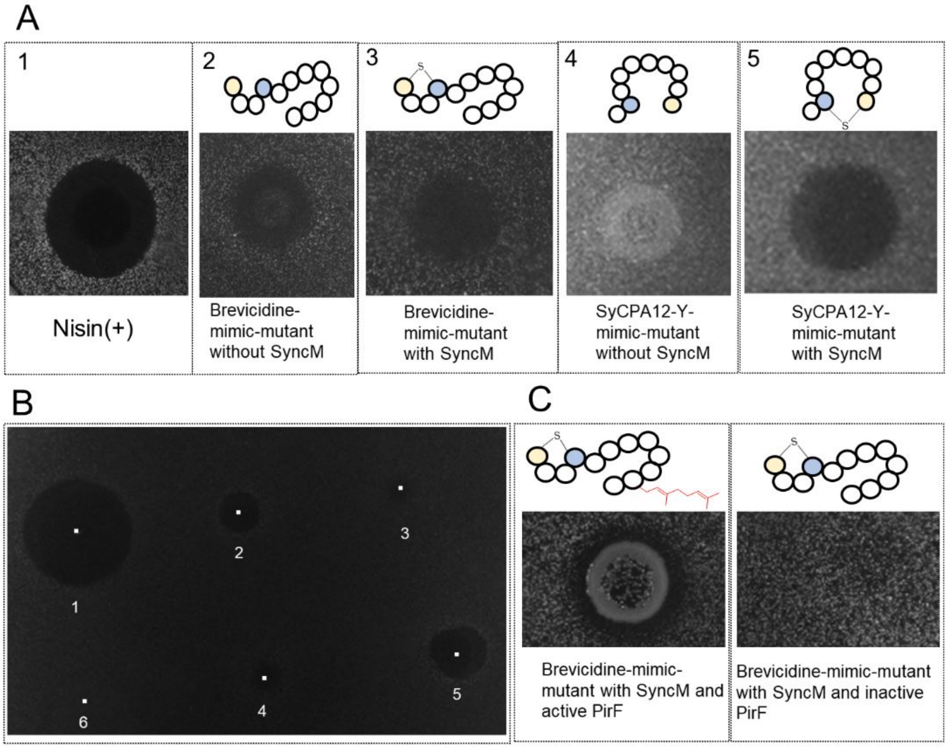

Figure S3. Agar diffusion antimicrobial activity assay against *B. subtilis 168* of the brevicidine-mimic-mutant and SyCPA12-mimic-mutant. Panel A displays the antimicrobial activity test results of modified cyclic peptides brevicidine-mimic-mutant and SyCPA12-mimic-mutant from the open C18 column purification in the presence of SyncM. Specifically, lanes 2 and 3 compare the activities of the linear peptide and the cyclic form of the Brevicidine-mimic mutant, while lanes 4 and 5 show the activities of

linear SyCPA12-mimic-mutant and its thioether macrocyclic ring-containing form. Panel B is the result of relative quantification of peptides prepared from HPLC by diameter of Inhibition zone. Panel C illustrates the activity test results for the lipidation of the cyclic peptide Brevicidine-mimic-mutant, comparing a reaction group treated with active PirF to a control group treated with PirF inactivated at 100 °C.

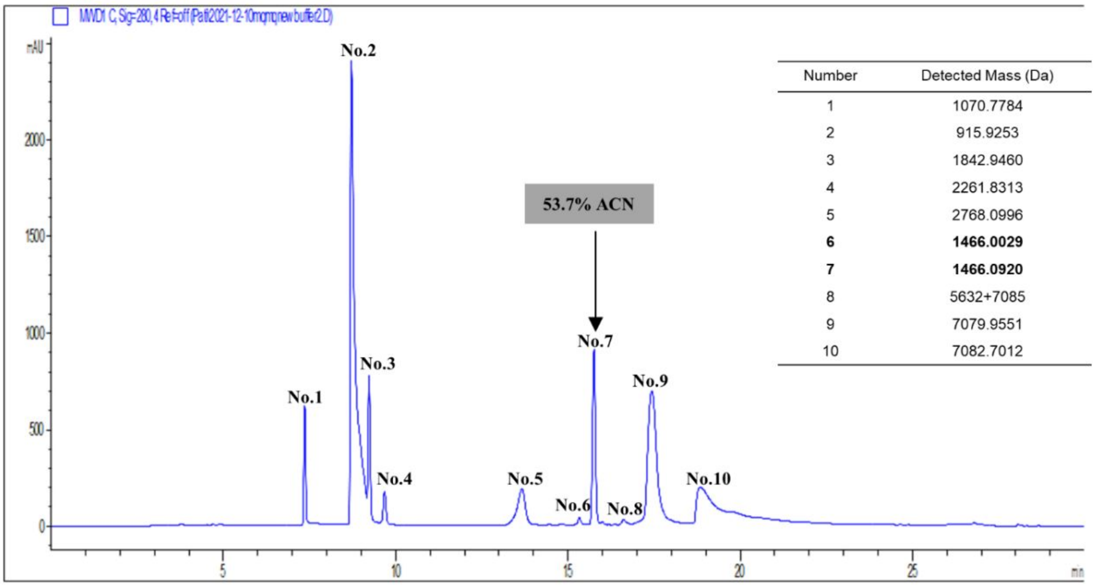

Figure S4. HPLC analysis and purification results of Brevicidine-mimic-mutant.

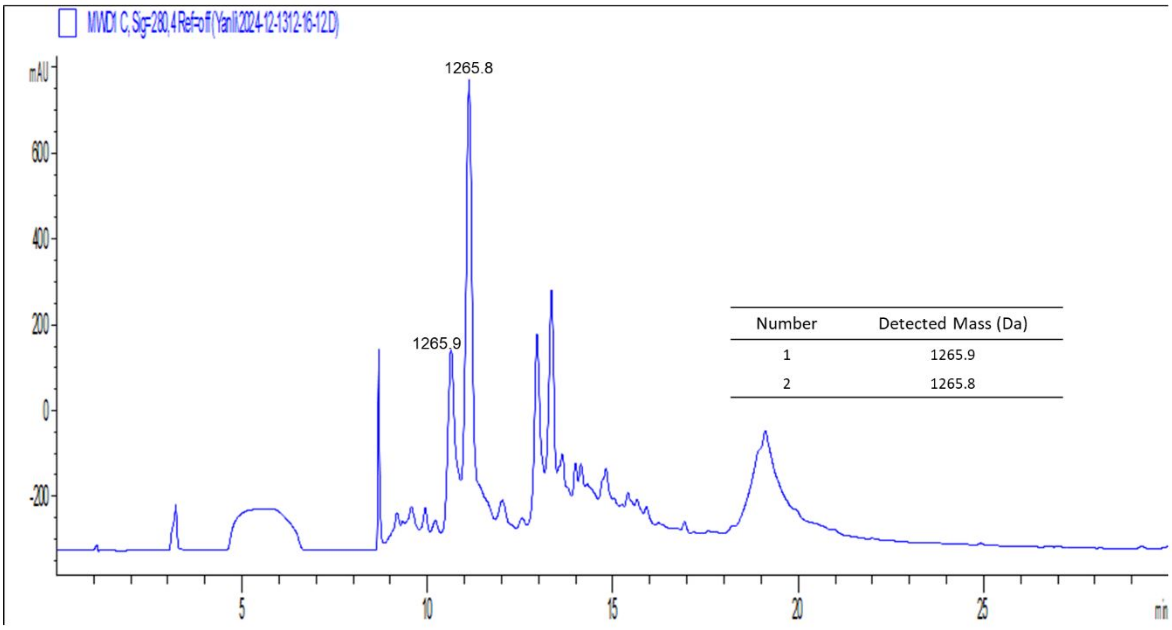

Figure S5. HPLC analysis and purification results of SyCPA12-mimic-mutant.

Nucleotide sequences of PirF：

ATGATCGTGGCGGAAATTCAGAAAAACAGTCTGAAAGAACAACGTATTAAATTTATTCGTAACCAC
CAGCAGGCATTCGATGTGGAACCAATTTACCCACTGCGTCTTTTTGAGGACTTCGTCATGAGTGT
GGAGGGCGACTGTAGTATTGAAGCCTCTTGCAAGATTGAACTGGACAAACTGATTGCATCCCGTT
TTATGCTGTTCTTCAAAGATCAGGAATGGGAGAAATACTTAACTCAGAGCTTAGCCTTTTTCGCC
AAGTCGAAAACCGCGTTGGTGTGCAGCTGGATTACTCATTATTGCAGAAATTTCTGGGCCATAATT
TTGATTTCTCCAAACTGGAAGTCTTATCCGCCGGGTTAGATCTGCGCACGAATTTAGCCGACAGC
AGCCTGAAAATTCATATCCGGATTAAAGATTATCCCGAAAAAATTAACCAGGCATTGTCACTCACT
ATCGATGGCGATGACCTGACAGCAGTACGTGATTTCTTAAGCGTTGTCGGGTTCGACTTCTATTTT
GACGGGCGCTCCGCAATTGAAATCTACCCGGAGGTTAAAGAAGAGGATTTTTTAAACCGAAGAC
CCAGGAGAAAGTGTGGCAGCACCTGCCAAAATTCGTTCTGGAACCTTTGCAGGTGACTAATCTGT
TTGGTTTCGGATTTAGCAAAACTAACCACAATCCCGTCGTTTACTATCGCCTCAAAGGCCGCCAG
GACCTGACGAACTATTTTAAAATCAATGATACAGCGCAGCGGGTGCATTCGTTTTACCAGCATCA
GGATATCCTGCCGAACATGTGGGTTGGGACCACCCAGAAAGAACTCGAAAAAACGCGCATCGAA
AATATCCGTCTGTACTACTATAAGTCATTCAAAATGGAGTAA

Amino acid sequence of PirF (35.08 kDa)

MIVAEIQKNSLKEQRIKFIRNHQQAFDVEPIYPLRLFEDFVMSVEGDCSIEASCKIELDKLIASRFMLFFK
DQEWEKYLTQSLAFFRQVENRVGVQLDYSLLQKFLGHNFDFSKLEVLSAGLDLRTNLADSSLKIHIRIK
DYPEKINQALSLTIDGDDLTAVRDFLSVVGFDFYFDGRSAIEIYPEVKEEDFFKPKTQEKVWQHLPKFV
LEPLQVTNLFGFGFSKTNHNPVVYYRLKGRQDLTNYFKINDTAQRVHSFYQHQDILPNMWVGTTQKE
LEKTRIENIRLYYYKSFKME*
